# Relationship between Item Responses of Negative Affect Items and the Distribution of the Sum of the Item Scores in the General Population

**DOI:** 10.1371/journal.pone.0165928

**Published:** 2016-11-02

**Authors:** Shinichiro Tomitaka, Yohei Kawasaki, Kazuki Ide, Maiko Akutagawa, Hiroshi Yamada, Toshiaki A. Furukawa, Yutaka Ono

**Affiliations:** 1 Department of Mental Health, Panasonic Health Center, Tokyo, Japan; 2 Department of Drug Evaluation and Informatics, Graduate School of Pharmaceutical Sciences, University of Shizuoka, Shizuoka, Japan; 3 Department of Health Promotion and Human Behavior, Kyoto University Graduate School of Medicine/School of Public Health, Kyoto, Japan; 4 Center for the Development of Cognitive Behavior Therapy Training, Tokyo, Japan; Chiba Daigaku, JAPAN

## Abstract

**Background:**

Several studies have shown that total depressive symptom scores in the general population approximate an exponential pattern, except for the lower end of the distribution. The Center for Epidemiologic Studies Depression Scale (CES-D) consists of 20 items, each of which may take on four scores: “rarely,” “some,” “occasionally,” and “most of the time.” Recently, we reported that the item responses for 16 negative affect items commonly exhibit exponential patterns, except for the level of “rarely,” leading us to hypothesize that the item responses at the level of “rarely” may be related to the non-exponential pattern typical of the lower end of the distribution. To verify this hypothesis, we investigated how the item responses contribute to the distribution of the sum of the item scores.

**Methods:**

Data collected from 21,040 subjects who had completed the CES-D questionnaire as part of a Japanese national survey were analyzed. To assess the item responses of negative affect items, we used a parameter *r*, which denotes the ratio of “rarely” to “some” in each item response. The distributions of the sum of negative affect items in various combinations were analyzed using log-normal scales and curve fitting.

**Results:**

The sum of the item scores approximated an exponential pattern regardless of the combination of items, whereas, at the lower end of the distributions, there was a clear divergence between the actual data and the predicted exponential pattern. At the lower end of the distributions, the sum of the item scores with high values of *r* exhibited higher scores compared to those predicted from the exponential pattern, whereas the sum of the item scores with low values of *r* exhibited lower scores compared to those predicted.

**Conclusions:**

The distributional pattern of the sum of the item scores could be predicted from the item responses of such items.

## Introduction

Depression is a common mental health disorder, with an estimated 350 million people of all ages affected around the globe [1]. Given that the presence of depressive symptoms is closely related to clinical levels of depression, there has been great interest in understanding the distribution of depressive symptoms in the general population [2, 3].

Several recent studies based on large sample sizes have shown that total depressive symptom scores in the general population follow an exponential pattern, except for the lower end of the distribution. In a data analysis on nearly 10,000 respondents to the British National Household Psychiatric Morbidity Survey, Melzer *et al*. observed that an exponential curve provided the best fit for total depressive and neurotic symptom scores on the Revised Clinical Interview Schedule (CIS-R) [4, 5]. The authors of the present study have similarly observed that the right tail of the distribution of total depressive symptom scores on the Center for Epidemiologic Studies Depression Scale (CES-D) follows an exponential curve, based on data on nearly 25,000 respondents to a national survey of the Japanese population [6]. A similar study also involving a large sample of Japanese employees further supported the exponential pattern of CES-D scores [7].

Although several recent studies based on large sample sizes have shown that total depressive symptom scores in the general population follow an exponential pattern, Melzer *et al*. has pointed out that total depressive symptomatic scores do not follow an exponential curve at specific levels of depressive symptom scores. Using the CIS-R, Meltzer *et al*. reported that total neurotic symptoms and depressive scores do not follow an exponential curve for symptom scores below 3 [5]. Furthermore, we performed a simulation study on depressive symptom scores modeled after the CIS-R and we found that the distribution of the simulated total depressive symptom scores did not follow an exponential pattern at the lower end of the distribution[8]. However, little research has been conducted on how non-exponential patterns occur at the lowest end of the distribution.

The CES-D allows an individual to self-rate the frequency of a variety of depressive symptoms (sadness, fatigue, etc.) on a scale consisting of four possible responses: “rarely (less than 1 day),” “some (1 to 2 days),” “occasionally (3 to 4 days),” and “most of the time (5 to 7 days)” (Radloff, 1997). Recently, we have shown that responses to each of the 16 individual items related to negative affect symptoms on the CES-D tend to exhibit exponential patterns for “some” and “most” responses in the general population, while this pattern is not observed for “rarely” responses[9]. These findings seem to suggest that the non-exponential item response pattern at the level of “rarely” responses may be related to the non-exponential patterns of total depressive symptom scores at the lower end of the distribution. To verify this hypothesis, we investigated how depressive symptom items, each of which has a different probability of “rarely” response, contribute to the distribution of total depressive symptom scores.

In the present study, we investigated the distribution of the sum of depressive symptom item scores in various combinations, using data from a large, cross-sectional national survey of the Japanese general population [10]. To assess the item responses in the range from “rarely” to “some,” we introduced a parameter *r* to denote the ratio of probability of “rarely” to the probability of “some.” According to the ranking of parameter *r*, the 16 negative items of the CES-D were grouped into combinations which consisted of 8 items each. Then, we compared the distributional patterns of the sum of 8 negative item scores on three combinations. Having confirmed that the distributional patterns of the sum of 8 negative item scores varied depending on the mean value of parameter *r*, the distributional patterns of the sum of 2 negative item scores, 4 negative items and 16 negative items were analyzed.

The goal of the present study was to determine whether the item responses in the range from “rarely” to “some” contribute to the non-exponential pattern of total scores at the lower end of the distribution and to examine whether the sum of negative item scores approximate an exponential pattern, except for the lower end of the distribution.

## Materials & Methods

The present study used data from the Active Survey of Health and Welfare (ASHW) conducted by the Japanese Ministry of Health, Labor, and Welfare in 2000 [10]. The ASHW is an annual nationwide survey conducted by the Japanese Government to collect the data required for policymaking and health promotion, in compliance with the Statistics Act. A legal and ethical approval of the ASWH was granted by the Japanese Ministry of Health, Labor, and Welfare. In 2000, the ASHW investigated depressive symptoms among a representative sample of the Japanese general population. To ensure that the sample was adequately representative, survey participants were selected from individuals aged 12 years and older, living across 300 communities in Japan. These communities were selected from 881,851 precincts identified in the 1995 Census using a stratified sampling design. The survey was conducted anonymously, and a verbal informed consent was obtained from all participants and legal guardians. The data and methods used in the survey have been described in detail in a previous report [10].

The questionnaire was returned by 32,729 respondents, even though the response rate was not published by the Ministry of Health, Labor, and Welfare and Health. However, the response rates for similar surveys conducted 3 and 4 years before were 87.1% and 89.6%, respectively [11]. Therefore, we assumed the response rate for the ASWH survey to be higher than 80%. A total of 707 participants who returned a blank questionnaire were excluded from the analysis.

The Japanese Ministry of Health, Labor, and Welfare examined our research program and allowed us to perform a secondary analysis on the anonymized data from the ASWH, in compliance with the Japanese Statistics Act. The present study was approved in 2014 by the ethics committee of the Panasonic Health Center (approval number 2014–1). The authors assert that all procedures contributing to this work comply with the ethical standards of the relevant national and institutional committees on human experimentation and with the Helsinki Declaration of 1975, as revised in 2008.

We excluded 1,394 respondents owing to the suspect validity of their responses (i.e., those who answered “rarely” or “most” for all items, regardless of the nature of the item). A total of 9,588 respondents were also excluded from the sample owing to missing information on one or more key study variables (i.e., depressive symptoms, age, sex). The final sample consisted of 21,040 respondents between 12 and 98 years of age (ages 12–19; N = 2457 [male; n = 1269], ages 20–29; N = 3748 [male; n = 1788], ages 30–39; N = 3761 [male; n = 1783], ages 40–49; N = 3629 [male; n = 1788], ages 50–59; N = 3569 [male; n = 1800], ages 60–69; N = 2253 [male; n = 1155], ages 70–79; N = 1161 [male; n = 517], ages 80–89; N = 412 [male; n = 108], ages 90–98; N = 50 [male; n = 15]).

### Measures

Depressive symptoms were assessed using the Japanese version of the CES-D [12]. This 20-item scale assesses the frequency of a variety of depressive symptoms experienced within the previous week (0 = rarely or never—less than 1 day, 1 = some or little time –1–2 days, 2 = occasionally or a moderate amount of times –3–4 days, and 3 = most or all the time –5–7 days), yielding a total score of 0–60 [13]. Higher scores indicate greater psychological distress. The 20 items of the CES-D are generally grouped into the following four subscales: depressive mood (items 3, 6, 9, 10, 14, 17, and 18); somatic symptoms (items 1, 2, 5, 7, 11, 13, and 20); interpersonal relations (items 15 and 19); positive affect (items 4, 8, 12, and 16). The positive affect items are reverse-scored.

In our previous work, we showed that the 16 negative items related to depressive mood, somatic symptoms, and interpersonal relations follow a common mathematical model, while the four items related to positive affect do not, suggesting that the items/symptoms associated with positive affect are not manifest variables of the unidimensional latent trait [9]. Thus, data on these 16 negative affect items were analyzed in the present study.

### Analysis procedure

To assess the item response in the range from “rarely” to “some,” the parameter *r*, which denotes the ratio of probability of “rarely” to the probability of “some,” was calculated for all the 16 negative items. A rank order was allocated according to the degree of parameter *r*. In addition, to assess the item response in the range from “some” to “occasionally,” the ratio of “some” to “occasionally” was calculated for all the 16 negative items. Using the parameter “r,” we compared the ratio of “rarely” to “some with the ratio of “some” to “occasionally.”

The distributions of the sum of negative affect items in various combinations were analyzed using log-normal scales. The fitting curve for an exponential model was estimated using least square method. The distributional patterns of the sum of 8 negative items, 4 negative items 2 negative items, and 16 negative items were compared among the different combinations. JMP Version 11 for Windows (SAS Institute, Inc., Cary, NC, USA) was used to calculate the descriptive statistics and the frequency distributions.

## Results

### Characteristics of item responses of 16 depressive symptoms

The item responses for the 16 negative affect items and the calculated parameter *r* are shown in Table 1. The value of parameter *r* varied depending on each item (mean ± S.D. = 4.07 ± 3.34).

**Table 1:** Item responses of participants (N = 21,040).

No.	Item	Response number (%)				Parameter r	Rate of “some” to “occasionally”	Rank order of r
		Rarely	Some	Occasionally	Most			
**1**	**Bothered**	10824 (51.4)	7492 (35.6)	2118 (10.1)	606 (2.9)	1.45	3.54	15
**2**	**Appetite**	14974 (71.2)	4322 (20.5)	1412 (6.7)	332 (1.6)	3.47	3.06	8
**3**	**Blues**	15063 (71.6)	4129 (19.6)	1256 (6.0)	592 (2.8)	3.65	3.29	6
**5**	**Concentrating**	10869 (51.7)	6821 (32.4)	2522 (12.0)	828 (3.9)	1.59	2.71	14
**6**	**Depressed**	11384 (54.1)	6216 (29.5)	2265 (10.8)	1175 (5.6)	1.83	2.74	12
**7**	**Effort**	9433 (44.8)	7988 (38.0)	2378 (11.3)	1241 (5.9)	1.18	3.36	16
**9**	**Failure**	11276 (53.6)	6345 (30.2)	2444 (11.6)	975 (4.6)	1.78	2.60	13
**10**	**Fearful**	16907 (80.4)	2892 (13.7)	849 (4.0)	392 (1.9)	5.85	3.41	3
**11**	**Sleep**	13234 (62.9)	4988 (23.7)	1920 (9.1)	898 (4.3)	2.65	2.60	11
**13**	**Talked**	13781 (65.5)	4919 (23.4)	1650 (7.8)	690 (3.3)	2.80	2.98	10
**14**	**Lonely**	16276 (77.4)	3110 (14.8)	1076 (5.1)	578 (2.7)	5.23	2.89	5
**15**	**Unfriendly**	17043 (81.0)	2913 (13.8)	748 (3.6)	336 (1.6)	5.85	3.89	2
**17**	**Crying**	19259 (91.5)	1283 (6.1)	351 (1.7)	147 (0.7)	15.01	3.66	1
**18**	**Sad**	15362 (73.0)	4277 (20.3)	982 (4.7)	419 (2.0)	3.59	4,36	7
**19**	**Dislike**	17235 (81.9)	2980 (14.2)	567 (2.7)	258 (1.2)	5.78	5.26	4
**20**	**Get going**	14933 (71.0)	4404 (20.9)	1083 (5.1)	620 (2.9)	3.39	4.07	9
**Average**		14241 (67.7)	4692 (22.3)	1476 (7.0)	630 (3.0)	4.07	3.40	

**Fig 1:**
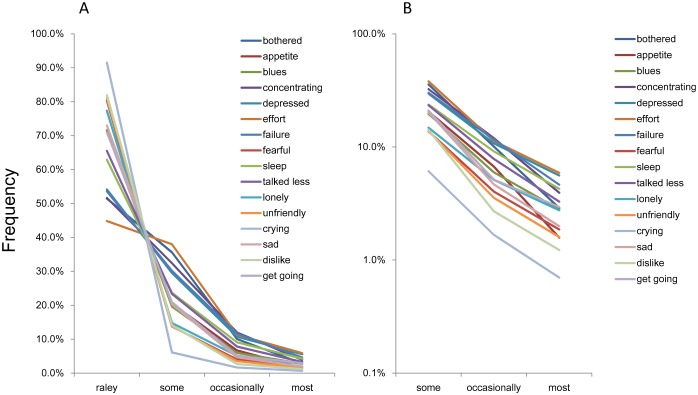
Item responses of the 16 negative affect items. The item responses of 16 negative affect items are presented on both normal (A) and log-normal (B) scales. (A) The item response for each of the 16 negative affect items showed a common pattern, which displays different patterns, with a boundary between “rarely” and “occasionally.” (B) The lines for the 16 items crossed each other between “rarely” and “occasionally,” whereas the same lines exhibited a right skewed pattern between “occasionally” and “most.” Using a log-normal scale, the item responses for the 16 items showed a linear pattern between “occasionally” and “most.”

As presented in Fig 1A, the item response for each of the 16 negative affect items showed a common trend, which presents different patterns and a boundary between “rarely” and “some.” As described in the previous report, the lines for the 16 items crossed each other between “rarely” and “some,” whereas the same lines exhibited a right-skewed pattern between “some” and “most” [9]. As shown in Fig 1B, the item responses for the 16 items showed a linear and parallel pattern between “some” and “most” using a log-normal scale, indicating that these 16 items exhibited an exponential pattern with the same parameter for this response level.

### Distributional patterns of the sum of 8 negative items

According to the rank order of parameter *r*, the 16 negative items of the CES-D were grouped into three combinations: high *r* group (item 2, item 3, item 10, item 14, item 15, item 17, item 18, and item 19), middle *r* group (item 2, item 3, item 6, item 11, item 13, item 14, item 18, and item 20), and low *r* group (item 1, item 5, item 6, item 7, item 9, item 11, item 13, and item 20). The high *r* group consists of any items from the first to the eighth in the rank order of *r*, the middle *r* group consists of any items from the fifth to the twelfth, and low *r* group consists of any items from the ninth to the sixteenth. The average of parameter *r* was 6.05 for the high *r* group, 3.33 for the middle *r* group, and 2.08 for the low *r* group.

**Fig 2:**
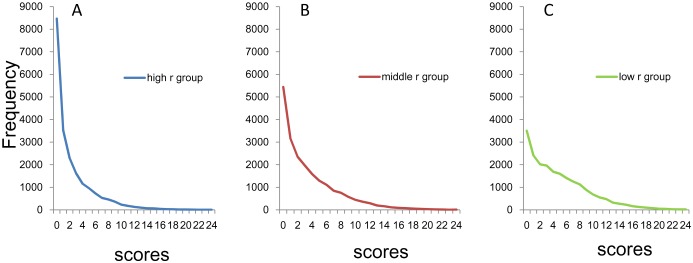
Distributions of the sum of 8 negative affect items. (A) High *r* group, (B) middle *r* group, and (C) low *r* group. While the distributions of the sum of 8 item scores for the three groups are commonly right-skewed, the frequencies of zero score were different across groups: The largest within the high *r* group (blue line), moderate within the middle *r* group (red line), and the smallest within the low *r* group (yellow line).

The distributions of the sum of 8 item scores for the three groups are shown in Fig 2A (high *r* group), Fig 2B (middle *r* group) and Fig 2C (low *r* group). While the distributions of the sum of 8 item scores for the three groups are commonly right-skewed, the frequencies of the zero score were different across groups.

**Fig 3:**
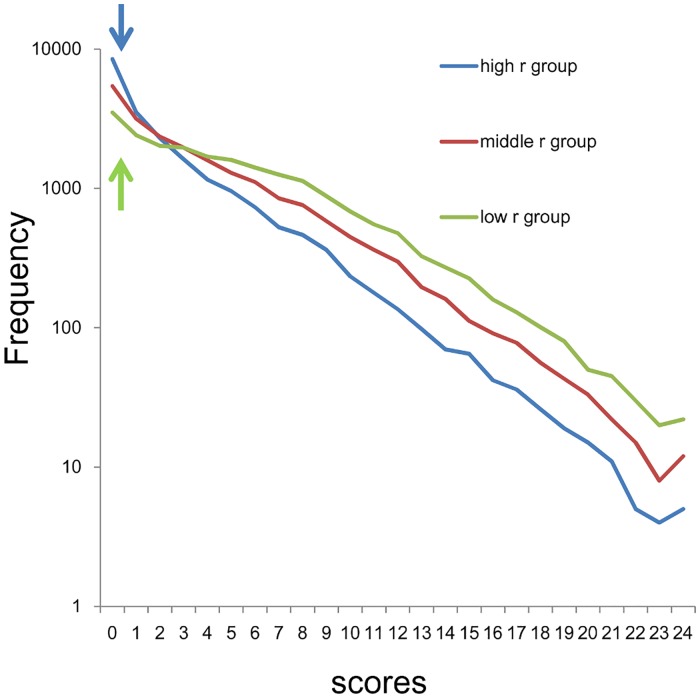
Distributions of the sum of 8 negative affect items using a log-normal scale. High *r* group (blue line), middle *r* group (red line), and low *r* group (yellow line). All three groups showed linear and parallel patterns from 3–8 scores to 20–23 scores. At the lowest end of the scores, the distribution for the high *r* group (blue line) exhibited higher frequencies compared to those predicted from the exponential pattern The distribution for the low *r* group exhibited lower frequencies compared to those predicted from the exponential pattern (yellow line). The distribution for the middle *r* group exhibited frequencies close to those predicted from the exponential pattern (red line).

Using a log-normal scale, all three groups showed linear and parallel patterns from 0–8 points to 24 points, suggesting that the sum of 8 item scores for the three groups followed an exponential pattern, with similar rate parameter (Fig 3). Conversely, as indicated by the arrows in Fig 3, the three groups exhibited individual patterns at the lower end of the distribution. While the distribution for the high *r* group showed higher frequencies compared to those predicted from the exponential pattern (blue arrow), the distribution for the low *r* group exhibited lower frequencies compared to those predicted from the exponential pattern (yellow arrow). Furthermore, the distribution for the middle *r* group exhibited frequencies close to those predicted from the exponential pattern.

The fitting curve using exponential model were calculated for data of high r group from 1–24 points (y = 4241e^-0.29x^, R^2^ = 0.99), middle r group from 0–24 points (y = 6456e^-0.26x^, R^2^ = 0.99) and low r group from 8–24 points (y = 1575e^-0.26x^, R^2^ = 0.99). Consistent with log-normal scale findings, exponential curve fitting showed a markedly higher coefficient of determination (R^2^ = 0.99) with similar rate parameter (-0.26 ~ -0.29).

### Distributional patterns of the sum of 4 negative items

To confirm the reproducibility of the findings observed for the sum of 8 items, we examined the distributions of the sum of 4 item scores. According to the parameter *r* ranking order, the 16 negative items of the CES-D were grouped into four combinations: high *r* group (item 10, item 15, item 17, and item 19), middle high *r* group (item 2, item 3, item 14, and item 18), middle low *r* group (item 6, item 11, item 13, and item 20), and low *r* group (item 1, item 5, item 7, and item 9). The high *r* group consists of any items from the first to the fourth in the rank order of *r*, the middle high *r* group consists of any items from the fifth to the eighth, the middle low *r* group consists of any items from the ninth to the twelfth, and the low *r* group consists of any items from the thirteenth to the sixteenth. The average of parameter *r* was 8.12 for the high *r* group, 3.98 for the middle high *r* group, 2.02 for the middle low *r* group, and 1.50 for the low *r* group.

**Fig 4:**
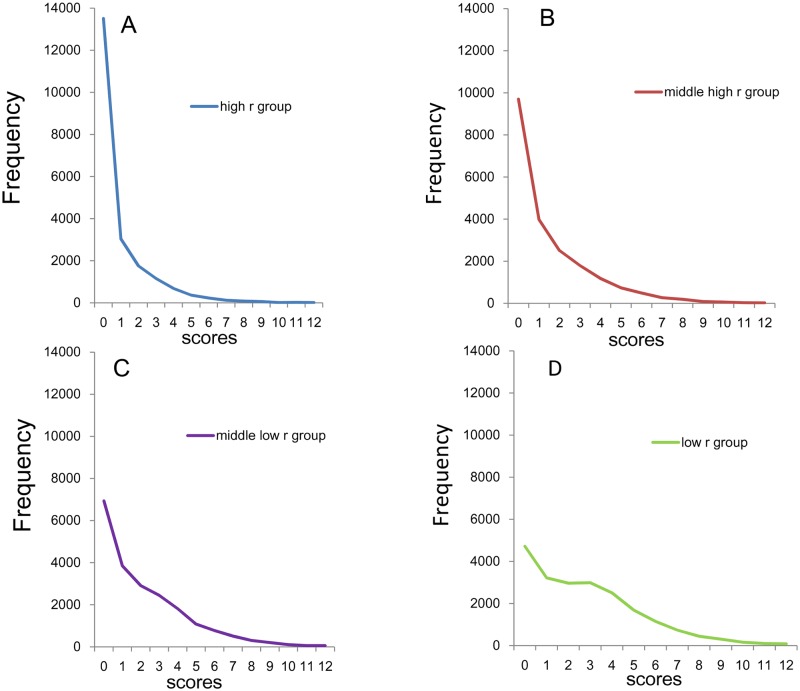
Distributions of the sum of 4 negative affect items. (A) High *r* group, (B) middle high *r* group, (C) middle low *r* group, and (D) low *r* group. While all distributions of the four groups are right-skewed, the low *r* group exhibited a plateau between score 1 and score 3.

The distributions of the sum of 4 item scores for the four groups are shown in Fig 4. While all distributions of the four groups are right-skewed, the low *r* group (Fig 4D) exhibited a plateau between point 1 and point 3.

**Fig 5:**
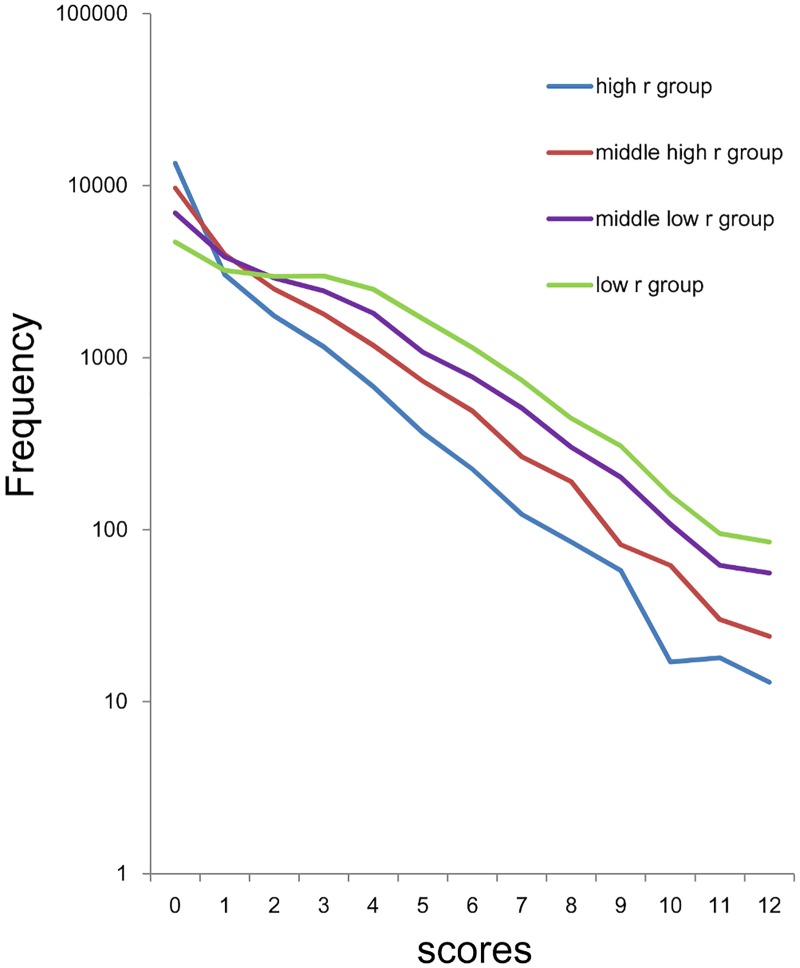
Distributions of the sum of 4 negative affect items using a log-normal scale. High *r* group (blue line), middle high *r* group (red line), middle low *r* r group (violet line), and low *r* group (yellow line). All four groups showed linear and parallel patterns from 1–3 scores to 11–12 scores. At the lowest end of the scores, the distribution for the high *r* group (blue line) and the middle high *r* group (red line) exhibited higher frequencies compared to those predicted from the exponential pattern, while the distribution for the middle low *r* group (violet line) and the low *r* group (yellow line) exhibited lower frequencies compared to those predicted from the exponential pattern.

Using a log-normal scale (Fig 5), all four groups showed linear and parallel pattern from 0–4 points to 12 points, consistent with the findings observed in the sum of 8 items. The four groups exhibited individual patterns under 1–3 points. At the lower end of the distributions, the distribution of the high *r* group (blue line) and middle high *r* group (red line) exhibited higher frequencies compared to those predicted from the exponential patterns, while the distribution of middle low *r* group (violet line) and low *r* group (yellow line) exhibited lower frequencies compared to those predicted from the exponential pattern. The divergence of the actual data from the predicted exponential pattern at the lower end of the distributions was especially evident in the high *r* group and low *r* group.

The curves of fit according to an exponential model were calculated for data of high r group from 1–12 points (y = 5063e^-0.52x^, R^2^ = 0.99), middle high r group from 0–12 points (y = 13037e^-0.49x^, R^2^ = 0.99), middle low r group from 0–12 points (y = 11341e^-0.41x^, R^2^ = 0.99) and low r group from 4–12 points (y = 4166.9e^-0.45x^, R^2^ = 0.99). Exponential curve fitting showed a markedly higher coefficient of determination in all four groups (R^2^ = 0.99) with similar rate parameter (-0.41 ~ -0.52).

### Distributional patterns of the sum of 2 negative items

Finally, we examined the distributions of the sum of 2 item scores. According to the parameter *r* ranking order, the 16 negative items of the CES-D were grouped into four combinations: high *r* group (item 15 and item 17), middle high *r* group (item 3 and item 14), middle low *r* group (item 6 and item 11), and low *r* group (item 1 and item 7). The high *r* group consists of the first and second item in the ranking order of parameter *r*, the middle high *r* group consists of the fifth and sixth, the middle low *r* group consists of the eleventh and twelfth, and the low *r* group consists of fifteenth and sixteenth. The average of parameter *r* was 10.43 for the high *r* group, 4.44 for the middle high *r* group, 2.24 for the middle low *r* group, and 1.31 for the low *r* group.

**Fig 6:**
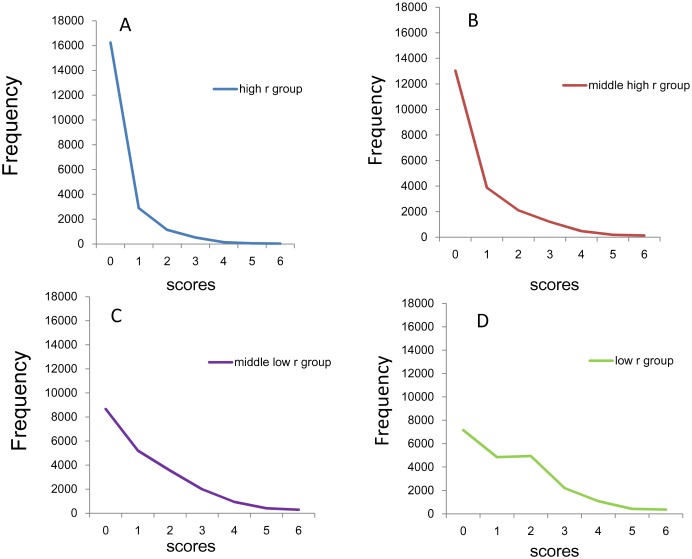
Distributions of the sum of 2 negative affect items. (A) High *r* group, (B) middle high *r* group, (C) middle low *r* group, and (D) low *r* group. While the distributions of the sum of 2 item scores for the four groups are right-skewed, the highest frequency of zero scores was different across groups.

The distributions of the sum of 2 item scores for the four groups are shown in Fig 6. While the distributions of the sum of 2 item scores for the four groups are right-skewed, the highest frequency of the zero score was different across groups.

**Fig 7:**
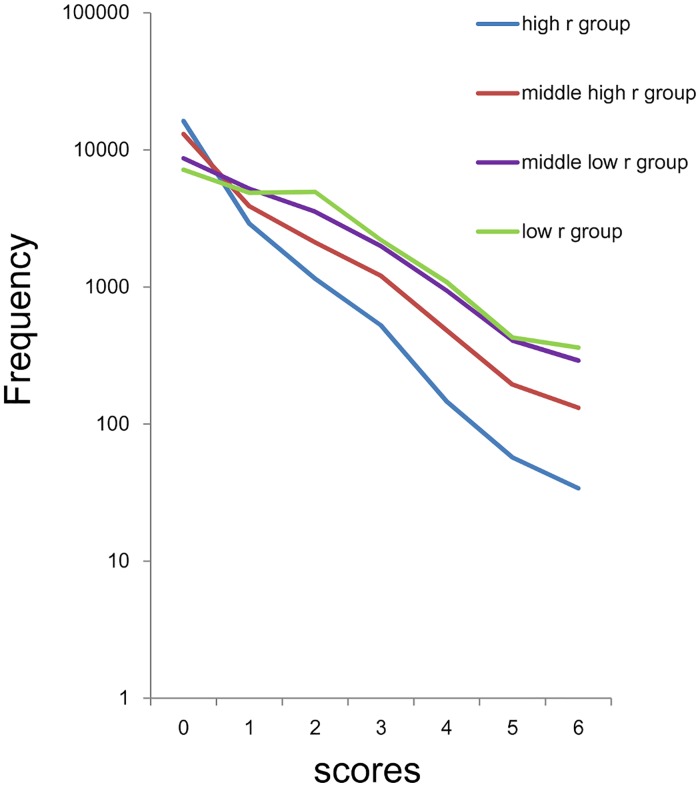
Distributions of the sum of 2 negative affect items using a log-normal scale. High *r* group (blue line), middle high *r* group (red line), middle low *r* group (violet line), and low *r* group (yellow line). All four groups showed linear patterns between score 2 and score 5. Between score 0 and score 2, the distribution for the high *r* group (blue line) and the middle high *r* group (red line) exhibited higher frequencies compared to those predicted from the exponential pattern, while the distribution for the low *r* group (yellow line) exhibited lower frequencies compared to those predicted from the exponential pattern. At score 6, all four groups exhibited higher frequencies compared to those predicted from the exponential pattern.

Using a log-normal scale (Fig 7), all four groups showed linear patterns from 1–2 points to 5 points, although the degree to which the sum of 2 items followed a linear and parallel pattern was not very clear compared to the patterns of the sum of 4 items and 8 items. From point 0 to point 2, the distribution for the high *r* group (blue line) and the middle high *r* group (red line) exhibited higher frequencies compared to those predicted from the exponential patterns, while the distribution for the low *r* group (yellow line) exhibited lower frequencies compared to those predicted from the exponential patterns. At point 6, all four groups exhibited higher frequencies compared to those predicted from the exponential patterns.

The curves of fit according to an exponential model were calculated for data of high r group from 1–6 points (y = 7263e^-0.93x^, R^2^ = 0.99), middle high r group from 1–6 points (y = 8594e^-0.72x^, R^2^ = 0.99), middle low r group from 1–6 points (y = 11041e^-0.62x^, R^2^ = 0.99) and low r group from 2–6 points (y = 8866e^-0.69x^, R^2^ = 0.97). Consistent with log-normal scale findings, although exponential curve fitting showed a higher coefficient of determination in all four groups (0.97–0.99), the rate parameter of the sum of 2 items (-0.62 ~-0.93) was not very similar compared to those of the sum of 4 items and 8 items.

### Distributional patterns of the total scores of 16 negative items

**Fig 8:**
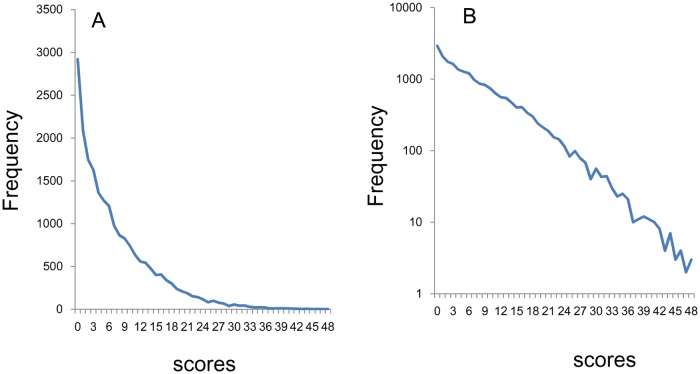
Distributional patterns of the total scores of 16 negative items. (A) The distribution of the total scores of 16 items is right-skewed. (B) Using a log-normal scale, the total scores of 16 items showed linear pattern from zero points to 48 points.

Finally, we examined the distributions of the total scores of 16 items. The average of parameter *r* was 4.07 for the 16 negative items. The distribution of the total scores of 16 items is right-skewed (Fig 8A). Using a log-normal scale (Fig 8B), the total scores of 16 items showed linear patterns from zero points to 48 points.

The curves of fit according to an exponential model were calculated for data of the total scores of 16 items (y = 3628e^-0.14x^, R^2^ = 0.99). Consistent with log-normal scale findings, exponential curve fitting showed a markedly higher coefficient of determination (R^2^ = 0.99).

## Discussion

The aim of the present study was to determine whether the item responses in the range from “rarely” to “some” contribute to the non-exponential pattern of total scores at the lower end of the distribution. The main findings of this study are as follows: (1) regardless of the choice of the items, the sum of negative item scores approximate an exponential pattern, except for the lower end of the distribution; (2) at the lower end of the distribution, the distributional pattern of the sum of the item scores varies depending on the parameter *r* of the chosen items, with the high parameter *r* group exhibiting higher frequencies compared to those expected from the exponential pattern, whereas the low parameter *r* group exhibits lower frequencies compared to those expected from the exponential pattern.

### The sum of negative item scores approximated an exponential pattern, except for the lower end of the distribution

Our findings indicate that the sum of negative item scores in various conditions approximates an exponential pattern, except for the lower end of the distribution. The reason why the sum of negative affect item scores approximates exponential patterns irrespective of their combination could be explained by a theory suggesting that negative affect items follow a unidimensional latent trait [6, 9]. According to this theory, negative affect items are manifest variables influenced by a unidimensional latent trait of depressive symptoms, and the latent traits of negative affect items follow an exponential distribution [7]. Furthermore, the simulation study using an ordinal scale model has confirmed that the sum of the ordinal scale items approximates a pattern similar to that of the latent trait distribution, except for the lower end of the distribution [8]. Taken together, these observations imply that the sum of negative affect item scores, using ordinal scales, approximates an exponential pattern in any combination.

Although the results of our study support the hypothesis that the latent traits of negative affect items follow an exponential distribution, the mechanism responsible for the exponential distribution of the latent traits is not clear. In general, an exponential distribution is observed where individual variability and total stability are organized together [14]. With respect to individual variability, personal mood and the related symptoms are generally the variables of interest. With regard to total stability, several studies have demonstrated the stability of depressive symptom scores in the general population [6, 15].

Analyzing the data of the second British National Survey of Psychiatry morbidity, Bebbington *et al*. demonstrated that selected affective symptoms scores related to paranoia followed an almost perfect exponential distribution [16]. From the viewpoint of the unidimensional latent trait, affective symptoms scores related to paranoia might have followed an exponential distribution because the items themselves were chosen from negative affective items. Further research is necessary to verify this speculation.

The rate parameters of the exponential models of the sum of negative affect item scores of 2 negative items, 4 negative items, 8 negative items and 16 negative items were -0.62 to -0.93, -0.41 to -0.52, -0.26 to -0.29, -0.14, respectively. The estimated parameters were similar across the groups with same number of items and increased as the number of summed items increased. These results suggest that the rate parameters of the exponential model of summed scores are associated with the number of items. Further mathematical explanation is necessary to elucidate the mechanism of the rate parameter variance.

### The sum of negative affect item scores exhibited a different distribution pattern at the lower end of the distribution

Our findings indicate that the distributional patterns of the sum of negative affect items varied depending on the parameter *r* of the selected negative items. The sum of negative affect items with high values of *r* exhibited higher frequencies compared to those predicted from the exponential pattern of total scores, whereas the sum of negative affect items with low values of *r* exhibited lower frequencies compared to those predicted from the exponential pattern of total scores.

The conditions that enable such findings can be speculated on. Whereas the sum of negative affect items in any combination approximates exponential patterns, with the same parameter, the number of subjects that corresponds to the range of the exponential pattern is different depending on the combinations of the items. The combinations of negative affect items with high values of *r* have a relatively small number in the range of the exponential pattern, whereas the combinations of negative affect items with low values of *r* have a relatively large number in the area of the exponential pattern. Since the total number of subjects is the same in all combinations, the sum of negative items with high values of *r* exhibits a relatively large number at the lowest end of the scores, whereas the sum of negative affect items with low values of *r* exhibits a relatively small number in the range of the exponential pattern, resulting in a different distribution pattern at the lowest end of the scores, depending on the mean value of *r*. It is worth noticing that, when the mean value of *r* is close to the ratio of “some” to “occasionally,” the distribution of the sum of negative affect items becomes similar to the predicted exponential pattern at the lowest end of the scores (Fig 3, red line, Fig 8B). These results suggest that the probability of “rarely” is a key index to predict the distributional pattern of the sum of negative affect items.

Analyzing the data of the British National Household Psychiatric Morbidity Survey, Meltzer *et al*. reported that there is a clear divergence between the actual data and the fitted exponential curve at the lower end of the distribution [5]. Since the CIS-R employs a binary method of item scoring (0–1: absence or presence), the mean probability of “absence” in item responses may contribute to the divergence of the actual data from the fitted exponential curve at the lowest end of the scores. In fact, according to our estimation, using data of the British National Survey of Psychiatry Morbidity, the mean probability of “absence” in CIS-R items (90.1%) is much higher than the probability of “rarely” in middle r groups, in the present study, and the actual data at the lower end of the distribution are higher than those predicted from the fitted exponential curve [15, 17].

### Strengths and limitations

The present study has some limitations. First, although we evaluated whether the sum of the item scores approximates an exponential distribution on a log-normal scale, we did not perform an analysis based on other mathematical models. In general, the most important part of model evaluation is testing whether the model fits empirical data better than other models. However, to the best of our knowledge, no other mathematical models for the sum of item scores have been reported so far. Thus, using graphical analysis and curve fitting, we performed the analysis limited to an exponential model. Future studies can evaluate the comparative fit of other models to our empirical data as reported in S1 File. Second, survey participants did not receive a standard psychiatric interview and the diagnosis associated with a structured interview. The present study did not encompass a diagnosis of depressive symptoms

Conversely, there is a methodological advantage in the present investigation. The sample was representative of the Japanese general population, which reduced selection bias. In addition, the large sample size (*N* = 21,040) enabled us to elucidate patterns in the distributions of depressive symptom items. Finally, the present study provides important information regarding the distribution of the sum of negative affect items, indicating that he parameter *r* of the chosen items could predict the distributional pattern of the sum of depressive symptom items in the general population. The degree to which the present findings can be generalized to empirical data is not clear yet, though further examination is warranted.

## Supporting Information

S1 File Raw data for distributions in Figs 2–7. This file includes raw data for distributions of the total depressive symptom scores in Figs 2–7.
(XLSX)
